# Baculoviral transduction facilitates TALEN-mediated targeted transgene integration and Cre/LoxP cassette exchange in human-induced pluripotent stem cells

**DOI:** 10.1093/nar/gkt721

**Published:** 2013-08-13

**Authors:** Haibao Zhu, Cia-Hin Lau, Sal-Lee Goh, Qingle Liang, Can Chen, Shouhui Du, Rui-Zhe Phang, Felix Chang Tay, Wee-Kiat Tan, Zhendong Li, Johan Chin-Kang Tay, Weimin Fan, Shu Wang

**Affiliations:** ^1^Department of Biological Sciences, National University of Singapore, 117543 Singapore, ^2^Department of Surgery, Program of Innovative Cancer Therapeutics, First Affiliated Hospital of Zhejiang University College of Medicine, 310009 Hangzhou, China and ^3^Institute of Bioengineering and Nanotechnology, 138669 Singapore

## Abstract

Safety and reliability of transgene integration in human genome continue to pose challenges for stem cell-based gene therapy. Here, we report a baculovirus-transcription activator-like effector nuclease system for *AAVS1* locus-directed homologous recombination in human induced pluripotent stem cells (iPSCs). This viral system, when optimized in human U87 cells, provided a targeted integration efficiency of 95.21% in incorporating a *Neo-eGFP* cassette and was able to mediate integration of DNA insert up to 13.5 kb. In iPSCs, targeted integration with persistent transgene expression was achieved without compromising genomic stability. The modified iPSCs continued to express stem cell pluripotency markers and maintained the ability to differentiate into three germ lineages in derived embryoid bodies. Using a baculovirus-Cre/LoxP system in the iPSCs, the *Neo-eGFP* cassette at the *AAVS1* locus could be replaced by a *Hygro-mCherry* cassette, demonstrating the feasibility of cassette exchange. Moreover, as assessed by measuring γ-H2AX expression levels, genome toxicity associated with chromosomal double-strand breaks was not detectable after transduction with moderate doses of baculoviral vectors expressing transcription activator-like effector nucleases. Given high targeted integration efficiency, flexibility in transgene exchange and low genome toxicity, our baculoviral transduction-based approach offers great potential and attractive option for precise genetic manipulation in human pluripotent stem cells.

## INTRODUCTION

Human pluripotent stem cells, as accessible and renewable human cell sources, have great prospective applications in developmental biology research, drug development, regenerative medicine and gene therapy. To realize the full potential of these stem cells, efficient and safe methods are required for precise genetic manipulation without affecting their self-renewal and differentiation capacities.

Gene targeting by homologous recombination (HR) is a technology that allows chosen DNA sequence to be genetically modified in a predetermined way. Given low efficiency of conventional HR in mammalian cells (1), zinc finger nuclease (ZFN)-induced DNA double-strand breaks (DSBs) have been used to stimulate endogenous HR machinery (2). More recently, nucleases based on transcriptional activator-like effectors (TALEs) have rapidly emerged as an alternative to ZFNs (3). TALEs are bacterial proteins characterized by a highly conserved central domain of tandem repeats that mediate binding to DNA. The DNA-binding specificity of each repeat unit is dictated by two hypervariable amino acids at positions 12 and 13 within the unit, called ‘repeat-variable di-residues’ (RVDs) (3). The 12th residue of a unit stabilizes the structural domain of protein backbone, whereas the 13th residue makes a base-specific contact with DNA (4,5). As one RVD recognizes one nucleotide of a DNA sequence, TALEs generated with new repeat combinations can recognize target sequences predicted by this simple DNA recognition code (3–6).

A transcription activator-like effector nuclease (TALEN) is a hybrid molecule that couples the DNA binding domain of a TALE protein with the catalytic domain of the FokI endonuclease (3). Customized TALENs have been successfully applied to target endogenous genes in a variety of organisms and stem cells, including human pluripotent stem cells (7). TALENs have rapidly developed into a chief next-generation technology for targeted genome editing owing to simplicity of design, essentially limitless targeting range (3,8–10), high targeting efficiency (4,8,11–16) and low genome toxicity (13,14).

Current TALEN approaches often use non-viral vector-based transfection methods to deliver genes encoding TALENs. Whether transduction of cells with more powerful viral vectors can be used to improve TALEN efficiency is yet to be assessed. Baculoviral vectors derived from the insect Autographa californica multiple nuclear polyhedrosis virus can efficiently transduce human embryonic stem cells (ESCs) and induced pluripotent stem cells (iPSCs) (17–20). Baculoviral vectors are capable of accommodating an additional 100 kb of DNA insert and can be used to transfer large and multiple DNA inserts (21,22). They replicate in insect cells but become replication incompetent in mammalian cells, a property that makes them easy for production and far less harmful to humans. When used for genetic modification of human cells, baculoviral vectors mediate transient transgene expression without integration into the host genome. This feature suits the purpose of temporary transgene expression well. In the context of TALEN-mediated targeted genetic engineering, only transient expression of the nuclease is required, whereas persistent nuclease expression and continuous enzymatic activity could lead to genomic instability and be harmful to host cells. Hence, baculoviral transduction could be a useful approach for TALEN technology. In support of the notion, a recent study has successfully used baculoviral vectors to deliver ZFNs and a DNA donor template for site-specific editing of the *CCR5* locus in human ESCs (23).

With the goal of developing an efficient method for site-specific transgene integration in human iPSCs, we tested in the current study a baculovirus-TALEN system for *AAVS1* locus-directed HR. The *AAVS1* locus is located within the gene encoding the protein phosphatase 1, regulatory (inhibitor) subunit 12C (PPP1R12C) on human chromosome 19 (19q13.3-qter) and is considered as a ‘safe harbor’ for addition of a transgene into human genome (24). *AAVS1* has an open chromatin structure flanked by insulator elements that shield a stably integrated transgene from *trans*-activation or repression (25). Robust and persistent transgene expression in human ESCs has been achieved with *AAVS1*-targeted integration (26,27). After TALEN-mediated transgene insertion into the *AAVS1* site, we further used a baculoviral vector-based Cre recombinase-mediated cassette exchange (Cre-RMCE) system (27) to replace the added transgene with a new transgene in the genetically engineered iPSCs. This combined approach offers the potential for successively loading and testing different transgenes at the same genomic site for functional gene analysis in iPSCs and for gene therapy applications of cells derived from iPSCs.

## MATERIALS AND METHODS

### Cell culture

Human embryonic kidney (HEK) 293 cells and human glioma U87 cells were cultured in Dulbecco’s modified Eagle’s medium (DMEM) (high glucose) medium with 10% fetal bovine serum (Invitrogen, Carlsbad, CA). Human foreskin fibroblasts (HFF, Millipore, Bedford, MA) were cultured in FibroGRO LS complete medium (Millipore). Human iPSCs were generated as described previously (20) and cultured on Matrigel-coated plates (BDscience, Franklin Lakes, NJ) with mTesR culture medium (STEMCELL Technology, Vancouver, BC, Canada) under a standard cell culture condition (37°C, 5% CO_2_) in a humidified incubator. The medium was changed daily, and iPSC colonies were passaged every 5–6 days with mechanical dissociation. For *in vitro* differentiation of iPSCs into embryoid bodies (EBs), iPSC colonies were detached by digestion with 0.1 mg/ml Dispase (STEMCELL Technology) at 37°C for 20–30 min, transferred to 6-well ultra-low attachment plates (Corning, Corning, NY) and cultured in suspension in DMEM/F12 (Invitrogen) supplemented with 20% KnockOut™ Serum Replacement (Invitrogen), l-glutamine (1 mM, Invitrogen) with trace amounts of β-mercaptoethanol solution and non-essential amino acids (Invitrogen). Medium was changed every alternate day for 2 weeks.

### Plasmids and recombinant baculoviral vectors

Left and right TALEN expression plasmids were constructed in pTAL.EF1α using custom TALEN service provided by Cellectis Bioresearch (Paris, France). The TALEN-coding sequences originating from a yeast expression plasmid were inserted into pTAL.EF1a using NcoI/HindIII. The left TALEN contains a nuclear Localization signal and a HA epitope, whereas the right TALEN contains a nuclear Localization signal and an S Tag epitope at the beginning of the N-terminal domain. The TALENs in the vectors are equipped with a wild-type FokI domain. As tested in yeast, the cleavage rate of the TALENs was 87% (Supplementary Figure S1).

pFastBac1 (Invitrogen), a donor plasmid that allows the gene of interest to be transferred into a baculovirus shuttle vector (bacmid) via transposition, was used as a plasmid backbone to construct recombination plasmids for baculovirus (BV) generation.

To construct pFB-TALEN, a human elongation factor-1α (EF1α) promoter from pEF1V5-HisA (Invitrogen) was introduced into pFastBac1 between BamHI and EcoRI, followed by inserting the right-TALEN plasmid with SpeI and XbaI. Using double-ligation approach, a 0.6 kb internal ribosome entry site (IRES) from pIRES (Clontech, Mountain View CA) and the left-TALEN were simultaneously subcloned into the vector using XbaI and HindIII. To construct the donor plasmid pFB-eGFP, an 810 bp left homologous arm and an 837 bp right homologous arm pertaining to the *AAVS1* locus were amplified from pZDonor-AAVS1 (Sigma-Aldrich, St Louis, MO) and inserted using SnaBІ/SalІ for the left arm and NotІ/BstBІ for the right homology arm into pFB-PGK-Neo-EGFP-LoxP, a pFastBac1 vector constructed in the laboratory previously (27), which contains heterospecific *loxP* sites. Then, a polycistronic cassette, containing the EF1α promoter, 4 iPSC transcription factor genes (human *Oct4*, *Klf4*, *Sox2* and *C-myc* genes joined with self-cleaving 2A sequence and IRES as a fusion gene), and the woodchuck hepatitis virus post-transcriptional regulatory element was amplified from pHAGE-EF1a-STEMCCA (Millipore, Bedford, MA) and inserted into pFB-eGFP using EcoRI/AscI to construct pFB-eGFP-4F. To construct pFB-mCherry, we replaced the *eGFP* gene with the *mCherry* gene in pFB-eGFP using SnaBІ/SalІ. Primers used for vector construction are listed in Supplementary Table S1. The construction of pFB-Cre was reported previously (27).

Recombinant BVs, including BV-TALEN, BV-eGFP, BV-eGFP-4F, BV-Cre and BV-mCherry, were generated using pFB-TALEN, pFB-eGFP, pFB-eGFP-4F, pFB-Cre and pFB-mCherry, respectively, and propagated in *Sf9* insect cells according to the protocol of the Bac-to-Bac Baculovirus Expression System from Invitrogen. Recombinant DNA research in this study followed the National Institutes of Health guidelines.

### T7 endonuclease I assay

TALENs induce sequence-specific DNA DSBs that can be repaired by the error-prone non-homologous end joining (NHEJ) system. The resulting DNA often contains small insertions or deletions (‘indel’ mutations) at a targeted genomic locus near the DSB site. These indel mutations can be detected *in vitro* by treating amplified DNA fragments with T7 endonuclease I (T7E1, 28). T7EI is a mismatch-resolving enzyme that can recognize heteroduplex DNAs and cleave DNA at single base pair mismatches, insertion and deletion. Thus, the T7EI assay has been used as a standard assay in the field to detect whether ZFNs or TALENs can cut endogenous target sequences in cells (28,29). Lower migration bands detected in the assay indicate genomic DNA disruption at the targeted locus. To perform the T7E1 assay, HEK293 cells were transfected with the plasmids using Lipofectamine™ 2000 Transfection Reagent (Invitrogen). Genomic DNA was extracted from treated cells with the DNeasy Blood and Tissue Kit (Qiagen, Hilden, Germany) 3 days later. A 742 bp fragment containing the TALEN cutting site within the *AAVS1* locus was amplified with the following primers: pForward 5′-GGGCATCTCTCCTCCCTCACCCAA-3′ and pReverse 5′-GATCCTCTCTGGCTCCATCGTAAGC-3′ using Platinum® PCR SuperMix High Fidelity (Invitrogen). The PCR products were purified with PCR purification kit (Qiagen) and then denatured, reannealed and digested with the mismatch-sensitive T7E1 (New England BioLabs, Beverly, MA). The fragments were separated with 2.0% agarose gel.

### γ-H2AX expression analysis and cell viability assay

To assess nuclease-associated genome toxicity, we monitored phosphorylation of histone H2AX (**γ**-H2AX), which correlates to DNA DSBs (30,31). Cells were transduced with BV-TALEN at an indicated multiplicity of infection (MOI) for overnight and harvested 3 days later for staining using anti-**γ-**H2AX (Abcam, Cambridge, UK) and goat anti-rabbit immunoglobulin G (IgG)–Fluorescein isothiocyanate (FITC) antibodies. The fluorescence intensity in the stained cells was recorded with image J for quantitation of **γ**-H2AX level per cell. The average **γ**-H2AX level in non-transduced control cells was defined as one. Cell viability was determined by CellTiter 96® AQueous Assay using 3-(4,5-dimethylthiazol-2-yl)-5-(3-carboxymethoxyphenyl)-2-(4-sulfophenyl)-2H-tetrazolium, inner salt (MTS, Promega, Madison, WI). The relative cell growth (%) compared with control cells was calculated by (absorbance of sample—absorbance of blank)/(absorbance of control—absorbance of blank) × 100%.

### TALEN-mediated HR and Cre-RMCE

For genetic modification of U87 cells, 2 × 10^4^ cells seeded on a 6-well plate were transduced with BV-TALEN and BV-eGFP at a MOI of 100 plaque forming units (pfu) per cell each. G418 selection at 400 µg/ml was started at day 3 post-transduction, and the medium was changed daily for 5 weeks. Single clones were then picked for further expansion in G418 selection medium. For genetic modification of iPSCs, 1 × 10^6^ cells were seeded on a 6-well plate coated with Matrigel 1 day before transduction. Next day, cells were incubated in medium containing BV-TALEN and BV-eGFP at an MOI of 100 pfu per cell each. The G418 selection at 20 µg/ml was started at day 3 post-transduction. After 10 days, EGFP-positive clones were selected for further expansion in G418 selection medium.

For Cre-RMCE, 2 × 10^6^ iPSCs cultured on Matrigel in mTeSR1 medium were transduced with BV-Cre at an MOI of 100 pfu per cell on day 4 following sub-culture. Twenty-four hours later, the medium was changed, and cells were again transduced with BV-mCherry at an MOI of 100 pfu per cell. Each colony was transferred to a single well of a Matrigel-coated 6-well plate and allowed to undergo clonal expansion through hygromycin B selection (25 μg/ml, AG Scientific, San Diego, CA) in mTeSR1 medium.

### PCR genotyping and Southern blot analysis

Genomic DNA of cells was isolated using DNeasy® Blood & Tissue Kit (Qiagen, Hilden, Germany). PCR genotyping was used to detect TALEN-mediated HR and Cre-RMCE events. PCR amplifications of genomic DNA were performed using the following parameters: an initial denaturation step at 94°C for 5 min followed by 35 cycles at 94°C for 15 s, 65°C for 45 s and 72°C for 150 s with a final extension step at 72°C for 10 min. Amplified products were analyzed on a 1% agarose gel.

For Southern blot analysis, genomic DNA (10 μg) was digested overnight with ApaLI. The digested DNA was loaded on a 1% agarose gel, and electrophoresis was performed for 10 h at 25V. Using the iBlot® Dry Blotting System (Invitrogen), DNA was then transferred to a positively charged nylon membrane. The membrane was washed twice with 1.5 M NaCl/0.5 M NaOH denaturing solution and air-dried. Ultraviolet cross-linking was performed at 130 mJ/cm^2^. The membrane was first pre-hybridized for 2 h and then hybridized overnight with Digoxigenin (DIG) Easy Hyb (Roche, Indianapolis, IN). DIG-labeled probes were synthesized using the PCR DIG Probe Synthesis Kit (Roche). Following hybridization, the membrane was first washed twice with 2 × SSC/0.1% SDS at 40°C and then twice with 0.1 × SSC/0.1% SDS at 65°C. Blocking and washing were performed using the DIG Wash and Block Buffer Set (Roche). The membrane was then incubated with an anti-digoxigenin-AP (DIG DNA Labelling and Detection Kit, Roche) that was detected by CDP-Star, ready-to-use (Roche). The membrane containing DNA was exposed to Chemiluminescent Image Analyzer (ImageQuant LAS 4000 mini, GE Healthcare Biosciences, Pittsburgh, PA) for 15 min. Primers used for PCR genotyping and for synthesizing DIG-labeled probes are listed in Supplementary Table S1.

### RT-PCR analysis

Total RNA was extracted from cells with RNeasy kit (Qiagen), and cDNA was synthesized using the SuperScript III First-Strand Synthesis System (Invitrogen). PCR amplifications were performed using the following parameters: an initial denaturation step at 94°C for 5 min followed by 35 cycles at 94°C for 15 s, 55°C for 45 s and 72°C for 45 s with a final extension step at 72°C for 10 min. Amplified products were analyzed on a 2% agarose gel. Primers used in RT-PCR analysis are listed in Supplementary Table S1.

### Immunofluorescence staining

Cells seeded on a Matrigel-coated, 24-well chamber slide were fixed in 4% paraformaldehyde for 30 min at room temperature. To induce the permeability of cells, 0.1% triton was added and incubated for 10 min. After washed with PBS, cells were incubated in a blocking solution (5% BSA) for 1 h. Primary antibodies used are those against OCT3/4 (Santa Cruz Biotechnology, Santa Cruz, CA), SOX2 (Abcam), Tra-1-60 (Santa Cruz Biotechnology) and SSEA4 (Santa Cruz Biotechnology). Goat anti-rabbit IgG–FITC or mouse anti-rabbit IgG-Rhodamine (Santa Cruz Biotechnology) was used as the secondary antibody. After antibody incubation, the samples were stained by DAPI (1:1000, Chemicon International, Temecula, CA) for nucleus staining. The images were then photographed by a fluorescence microscope.

## RESULTS

### Cellular effects of BV-TALEN

**Figure 1.**
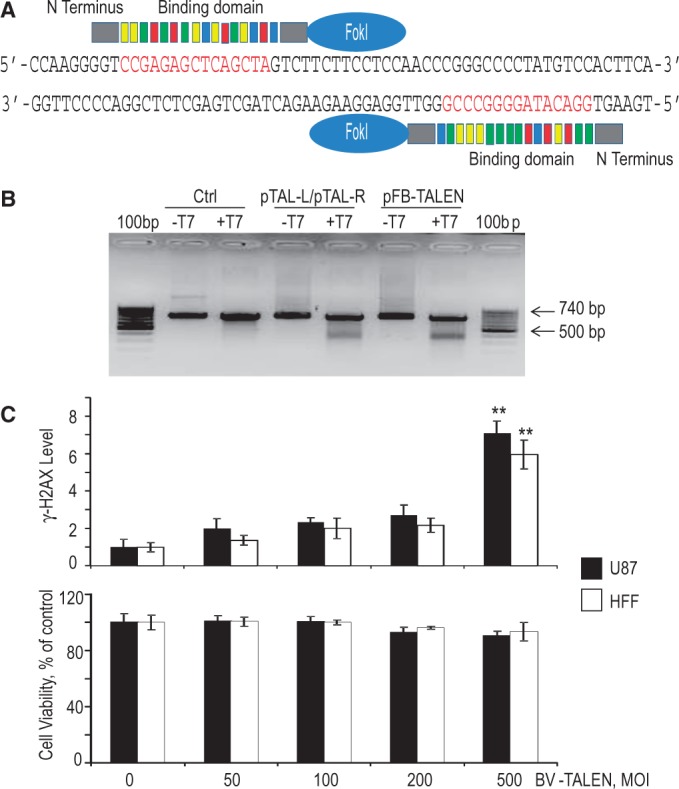
BV-TALEN and its potential cytotoxic effects. (**A**) Schematic representation of the *AAVS1*-TALEN constructs and the binding sites for the *AAVS1*-TALEN pairs. FokI: FokI endonuclease. The C terminus of TALEN recognition sequence is fused with the FokI cleavage domain. The DNA sequences represent part of the *AAVS1* locus and include TALEN-binding sites (red letters). The DNA binding domains of TALEN are colored to indicate the identity of the RVD. Color, RVD and its cognate targeted DNA base: Red = NI = A, Yellow = HD = C, Green = NN = G. and Blue = NG = T. (**B**) Target gene disruption by TALEN-expressing plasmid vectors. HEK293 cells were either co-transfected with pTAL-L and pTAL-R or transfected with pFB-TALEN, an expression vector in which the two TALEN arms were cloned into one plasmid vector. The lower migrating bands indicate the TALEN-mediated gene disruption. 100 bp: 100 bp molecular marker. +T7 and −T7: Incubation with or without T7E1 endonuclease. (**C**) Quantitation of **γ-**H2AX level per cell (top) and cell viability assay (bottom). Human U87 glioma cells and HFF were transduced with BV-TALEN at an indicated MOI overnight. Top: Immunostaining of the transduced cells was performed 3 days later using anti-**γ-**H2AX to assess nuclease-associated genome toxicity. Bars: SE. **P < 0.01 versus the control (BV-TALEN 0) group by analysis of variance. Bottom: TALEN-related cytotoxicity was analyzed by measurement of cell viability in MTT assay at day 3 post-transduction.

TALENs designed to target the *AAVS1* locus were synthesized using a commercial service. The TALENs are chimeric proteins consisting of *AAVS1*-specific DNA binding domains fused to the non-specific cleaving endonuclease FokI (Figure 1A)*.* To confirm the TALEN-induced cleavage at the endogenous target *AAVS1* site, the mismatch-sensitive nuclease assay using the T7E1 endonuclease was performed on genomic DNA from HEK293 cells transfected with TALEN-expressing plasmid vectors. As shown in Figure 1B, a portion of the targeted DNA collected from the treated cells was digested into small fragments ∼350–390 bp in size. Although TALEN cleavage rate was not quantified, the intensity of the small fragments from the cells transfected with pFB-TALEN, an expression vector in which the two TALEN arms were inserted into one plasmid vector, appeared higher than that from the cells co-transfected with two plasmid vectors expressing left and right TALEN separately. These results demonstrate that the TALENs can induce DSBs at *AAVS1* in human genome.

We then used pFB-TALEN to produce a baculoviral vector, BV-TALEN. As TALEN-mediated gene editing is based on DNA DSB-stimulated HR, we assessed genome toxicity associated with TALEN-induced DSBs, which might occur when high levels of TALENs are expressed following high-efficient baculoviral transduction. We transduced human U87 and HFF cells using BV-TALEN alone, without any donor DNA template, and analyzed the cells using a well-validated assay with antibody against phosphorylated histone H2AX (**γ**-H2AX) to visualize DNA DSBs. **γ**-H2AX is generated in response to DNA damage and forms foci at DSBs (14,30,31). As revealed by quantitation of **γ**-H2AX level per cell, BV-TALEN at an MOI of 200 pfu per cell or lower did not significantly increase DNA cleavage in the tested cells (Figure 1C). Cell viability assay revealed an increase in cell death, although not statistically significant, only after BV dose increased to an MOI of 200 or 500 pfu per cell (Figure 1C). Based on the aforementioned findings, transduction using BV-TALEN at MOI of 100 pfu per cell was selected for the following studies.

### Targeted gene addition in human U87 glioma cells by BV-TALEN

**Figure 2.**
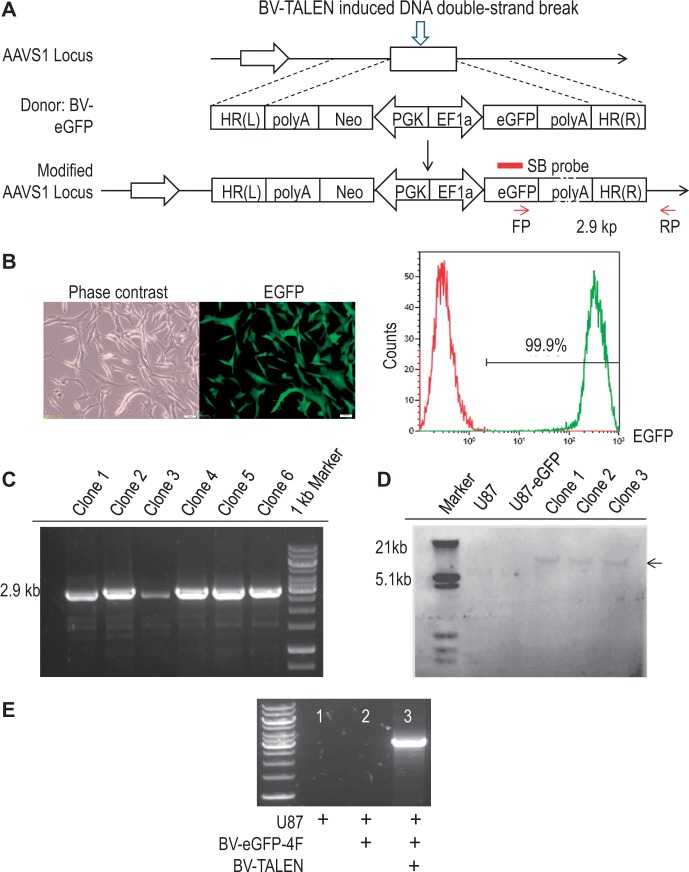
HR trigged by BV-TALEN in U87 cells. (**A**) Schematic diagram showing *AAVS1*-locus integration of the *Neo-eGFP* expression cassette provided by the DNA donor BV-eGFP following BV-TALEN-induced DNA DBS. HR(L) & HR(R): Left and right arms for HR. FP & RP: Binding sites for PCR forward and reverse primers. SB probe: The probe binding site used for Southern blot analysis. (**B**) Stable EGFP expression in U87 cells. Left: Representative phase and fluorescence images. Right: Flow cytometry analysis to determine the percentage of EGFP-positive cells 3 months after BV transduction and maintained without G418. Bar = 50 µm. (**C**) *AAVS1*-specific transgene integration. The amplification of a 2.9 kb fragment is used to identify gene insertion at the *AAVS1* locus. (**D**) Southern blot analysis to detect the modified *AAVS1*. Following digestion with ApaLI and hybridization, single 12 kb fragment (arrow) was detected in three transgenic clones opposed to no fragment detected in wild-type U87 cells. (**E**) TALEN-mediated HR of a 13.5 kb DNA insert. U87 cells were transduced with a BV vector containing a 13.5 kb *eGFP*-4F donor template alone or co-transduced with this vector and BV-TALEN. PCR analysis demonstrates the co-transduction-mediated, site-specific integration of the 13.5 kb donor template at the *AAVS1* locus.

To test targeted gene addition into the *AAVS1* locus, we generated another BV vector, BV-eGFP, with an expression cassette carrying the *eGFP* gene under the control of the EF1α promoter and flanked on both sides by sequences homologous to the *AAVS1* locus. As illustrated in Figure 2A, the process for BV-TALEN-mediated *AAVS1* targeted integration of the *eGFP* expression cassette comprises two steps. First, the TALEN arms expressed from BV-TALEN bind on their *AAVS1* target sequence to induce a DNA DSB by the catalytic domain of FokI endonuclease. Then, the DSB is repaired by HR machinery using BV-eGFP as a donor template. We first used human U87 cells, a cell line that can be transduced by baculoviral vectors efficiently, to optimize the baculovirus-mediated targeted transgene insertion. Cells were co-transduced with BV-TALEN and BV-eGFP and then subjected to G418 selection for 5 weeks. The survived cells became EGFP-positive and exhibited persistent EGFP expression for at least 3 months without G418 (Figure 2B). PCR genotyping was performed in 41 clones derived from single cell cloning in three individual experiments and demonstrated that the mean efficiency of site-specific integration of the *eGFP* donor cassette into the *AAVS1* locus was 95.21% (Figure 2C, Supplementary Figure S2; Table 1). To verify the possible existence of multiple transgene copies in the transgenic clones, Southern blot analysis was performed on eight transgenic clones using a probe specific for the *eGFP* gene (Figure 2D; Supplementary Figure S3). The detection of a 12 kb fragment confirmed the integration of the *eGFP* donor cassette within the *AAVS1* locus*.* We were unable to detect fragments of other sizes, confirming the absence of random integration. Putting together, these results indicate a high site-specific integration efficiency offered by our baculovirus-TALEN system.

**Table 1.** Targeting efficiency of TALEN-mediated HR in U87 cells

Exp.	No. of G418- resistant clones	No. of analyzed clones	Targeted integration (# clones)	Targeting efficiency (%)	Mean targeting efficiency (%)
1	20	13	12	92.31	
2	25	15	14	93.33	
3	21	13	13	100	95.21

The size of the *Neo-eGFP* cassette is 5 kb. To test whether a larger cassette can be integrated in a site-specific manner using a baculovirus-TALEN system, we constructed a polycistronic baculoviral vector, BV-eGFP-4F, with a 13.5 kb donor cassette that contains *Oct4-Klf4-Sox2-cMyc* multiple transgenes flanked by right and left homologous arms pertaining to the *AAVS1* locus. Seven days following co-transduction with this virus and BV-TALEN in U87 cells, the genomic DNA of transgenic cells was isolated and subjected to PCR genotyping by using a primer specific for the woodchuck hepatitis virus post-transcriptional regulatory element sequence present in the donor vector and a primer specific for the *AAVS1* locus in chromosome 19 downstream of the 3′-end of the right homologous arm. The amplification of a 3 kb fragment confirmed the successful site-specific integration of the donor cassette into the *AAVS1* locus by TALEN-mediated HR (Figure 2E). Moreover, no integration was detected in wild-type U87 cells or U87 cells transduced with BV-eGFP-4F alone, without the use of BV-TALEN. Genome insertion of the *eGFP*-4F donor cassette could be useful in future for efficient cellular reprograming of human somatic cells.

### Genetic characterization of NHEJ mutations in genetically modified U87 clones

In addition to resulting in HR with donor DNA, DNA DSB induced by TALENs can also be repaired by NHEJ without the involvement of a homologous template, which can maintain the wild-type sequence, or introduce loss or gain in base pairs. We performed DNA sequencing in eight monoallelically modified U87 clones to determine the frequency of NHEJ mutations at the putative wild-type *AAVS1* locus on the remaining allele without the *eGFP* cassette integration. We observed only one clone containing a 3-bp deletion and seven of eight clones being wild-type at the site (Table 2). A potential limitation of TALEN technology is the induction of off-target DNA breaks at related sequences throughout the host cell genome. Using the Basic Local Alignment Search Tool, eight genome sites highly similar to the *AAVS1* TALEN-binding sequences were identified as the most probable off-target cleavage sites by BV-TALEN (Supplementary Table S2). These eight sites were amplified from genomic DNA isolated from wild-type U87 and 3 *AAVS1*-targeted U87 clones and sequenced. As the PCR products were from a single-cell cloning derived, homogenous cell population in each clone, mutations in the off-target sequence will show as single or double peaks that are different from reference sequence. DNA sequencing of the PCR products revealed no evidence of deletions or insertions at these off-target sites, and all sites were wild-type (Supplementary Figure S4). Thus, co-transduction with BV-TALEN and BV-eGFP resulted in the targeted addition of the *eGFP* cassette into the genomic TALEN target site with high specificity.

**Table 2.** Analysis of NHEJ mutations in U87 cells

Reference seq	TCCGAGAGCTCAGCTAGTCTTCTTCCTCCAACCCGGGCCCCTATGTCCA
Clone 1	TCCGAGAGCTCAGCTAGTCTTCT - - - TCCAACCCGGGCCCCTATGTCCA
Clones 2–8	TCCGAGAGCTCAGCTAGTCTTCTTCCTCCAACCCGGGCCCCTATGTCCA

NHEJ mutations at the remaining putative wild-type *AAVS1* locus on the allele without integration of the *eGFP* expression cassette were analyzed. The *AAVS1* locus was amplified from genomic DNA isolated from eight EGFP-positive U87 clones generated from single cell cloning for DNA sequencing. The reference sequence of the analyzed site and DNA sequencing results from the eight clones are listed. Only one deletion was observed in Clone 1.

### Targeted gene addition in human iPSCs by BV-TALEN

**Figure 3.**
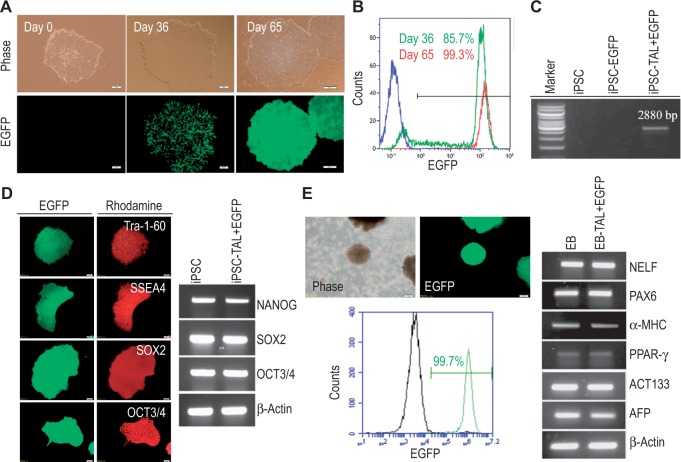
BV transduction mediated *AAVS1*-directed HR in human iPSCs. (**A**) EGFP expression in *AAVS1*-targeted iPSC clones. iPSCs were transduced with BV-TALEN and BV-eGFP and subject to G418 selection. Fluorescence and phase contrast images of iPSC colonies on day 0, 36 and 65 post-transduction are shown. Pure EGFP-positive colonies were obtained after G418 selection for ∼60 days. Bar = 50 µm on day 0 and 36, and = 200 µm on day 65. (**B**) Flow cytometry analysis to examine the purity of EGFP-positive iPSC colonies. Green color: iPSC colonies collected on day 36; Red color: iPSC colonies collected on day 65. (**C**) PCR genotyping to confirm the *AAVS1* locus integration of the *eGFP* cassette. Genomic DNA was extracted on day 7 post-transduction from original, wild-type iPSCs (iPSC), iPSCs transduced with BV-eGFP alone (iPSC-EGFP) and iPSCs co-transduced with BV-TALEN and BV-eGFP (iPSC-TAL + EGFP). (**D**) Maintenance of pluripotency in transgenic iPSCs. Left: Immunostaining to detect the expression of pluripotency markers. Bar = 50 µm. Right: RT-PCR to detect pluripotent marker gene expression. (**E**) Persistent transgene expression maintained after iPSC differentiation. Left, top: Formation of EBs and EGFP expression in EBs. Bar = 50 µm. Left, bottom: Flow cytometry analysis to quantify the EGFP-positive cells in EBs. Right: RT-PCR analysis to detect the expression of three germ lineage markers in EBs.

Encouraged by the results from human U87 cells, we moved forward to test BV-TALEN-mediated site-specific integration in iPSCs derived from human HFF (20). iPSCs were cotransduced with BV-TALEN and BV-eGFP, and G418 selection started 48 h later. After selection for 2 weeks, iPSC colonies with EGFP-positive cells were mechanically selected and expanded further under G418 selection. As shown in Figure 3A, pure EGFP-positive iPSC colonies were obtained after propagation for 2 months. The percentage of eGFP positive cells in these colonies was 85.7% on day 36 and became 99.3% by day 65 (Figure 3B). We also confirmed that the transduction of iPSCs with BV-eGFP alone yielded no colonies through G418 selection (unpublished observation), suggesting that BV-TALEN is necessary for insertion of the *eGFP* gene into iPSC genome.

PCR genotyping using genomic DNA of the pure EGFP-positive iPSC colonies confirmed the successful site-specific integration of the transgene cassette within the *AAVS1* locus by TALEN-mediated HR by amplification of a fragment ∼2.9 kb (Figure 3C). The colonies with strong and persistent EGFP expression could be maintained over 40 passages for at least 8 months, confirming that EGFP expression in the selected clones was stable. Immunostaining assay and RT-PCR analysis further demonstrated that these EGFP-positive iPSCs continued to express stem cell pluripotency markers (Figure 3D) and maintained the ability to differentiate into three germ lineages in transgenic iPSC-derived embryoid bodies (Figure 3E), indicating that their pluripotent stem cell properties and differentiation capacities were not compromised by baculoviral transduction and TALEN-mediated genetic modification. We also observed persistent expression of EGFP in differentiated progenies in the embryoid bodies. Quantification by flow cytometric analysis indicated that 99.7% of cells the embryoid bodies were EGFP-positive (Figure 3E). Karyotyping analysis of the transgenic EGFP-positive iPSCs demonstrated that there was no numerical or structural aberration, and the cells retained a normal karyotype (Supplementary Figure S5).

### Cre-RMCE following TALEN-mediated transgene insertion

**Figure 4.**
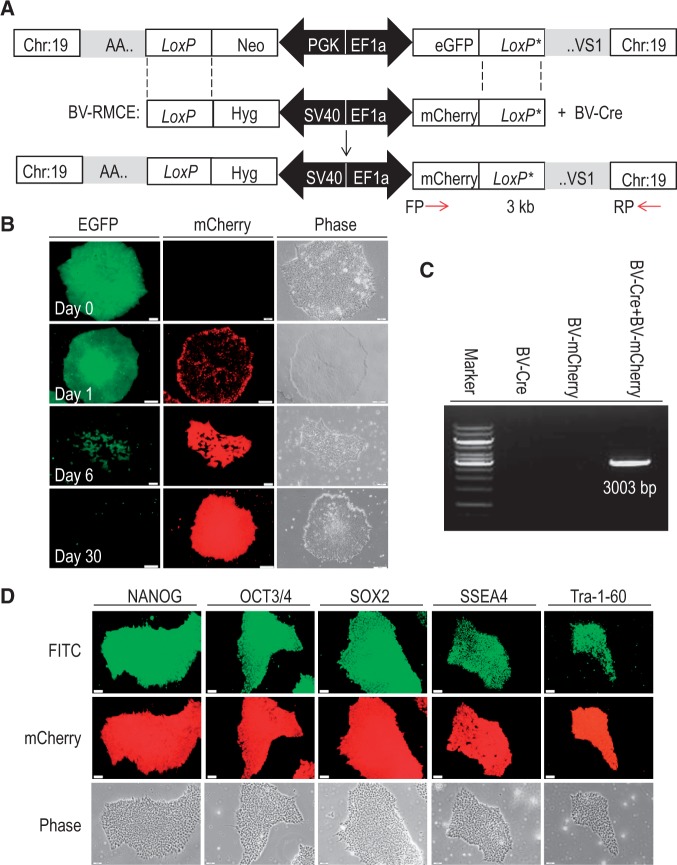
BV transduction-based Cre-RMCE (BV-RMCE) in EGFP-positive iPSCs. (**A**) Schematic representation of replacing the *eGFP* gene with the *mCherry* gene through BV-RMCE at the *AAVS1* locus. Both constructs were flanked by the same heterospecific *loxP* sequences that permit cassette exchange in the presence of Cre recombinase expressed from BV-Cre. (**B**) Fluorescence and phase contrast imaging to show the increase in mCherry expression under hygromycin selection at different intervals of time following BV-RMCE. Short bar = 50 µm and long bar = 200 µm. (**C**) PCR genotyping to confirm *AAVS1* integration of the *mCherry* cassette. A primer specific for the *mCherry* gene and a primer specific for chromosome 19 downstream of the 3′-end of the right *AAVS1* homologous arm were used. The amplification of a 3-kb fragment demonstrates the successful cassette exchange at *AAVS1* through BV-RMCE. (**D**) Maintenance of pluripotency of mCherry-positive iPSC colonies. FITC secondary antibodies were used for immunostaining to detect expression of pluripotent markers. Bar = 50 µm.

Cre-RMCE is another powerful tool to mediate integration of a transgene into a predefined genomic site. Different from TALEN technology that involves the introduction of DNA DSB, Cre cleaves one DNA strand at a time at a target point and triggers a site-specific recombination reaction that causes exchange between specific *loxP* sites (1). We have previously adopted a two-step genetic modification process using HR followed by RMCE to direct transgene integration into the *AAVS1* locus in hESCs and demonstrated that baculoviral transduction is efficient in supporting integration of a transgene expression cassette into a target site embedded into the hESC genome (27). In the present study, we have designed our insertion cassette in the TALEN donor vector to be flanked by a pair of heterospecific *loxP* sequences to facilitate Cre-RMCE. We then tested whether the transgene inserted through TALEN technology could be replaced by another transgene using Cre-RMCE (Figure 4A).

**Figure 5.**
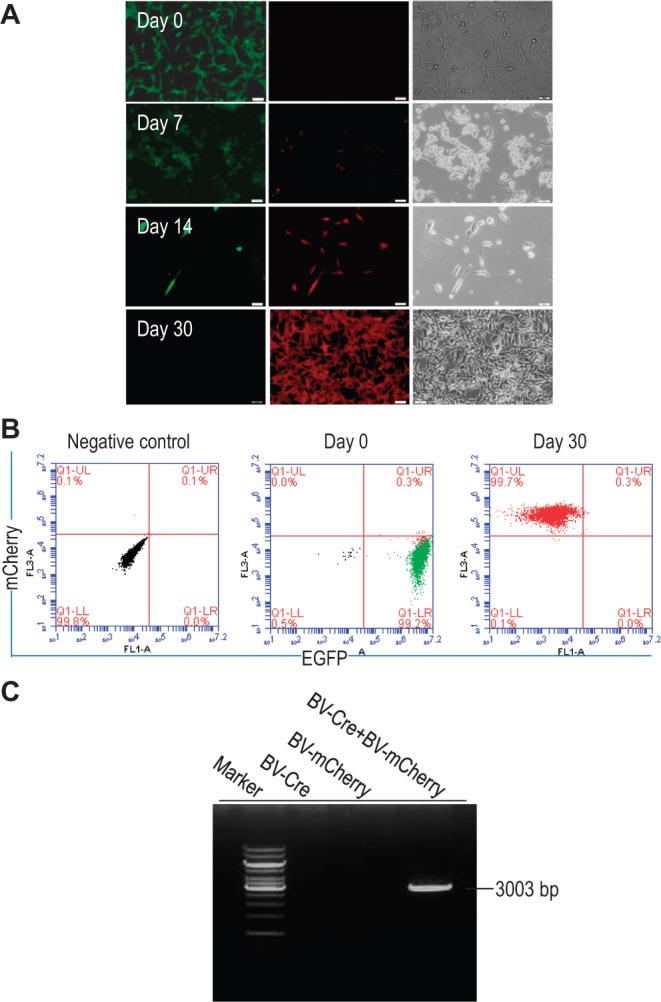
BV transduction-based Cre-RMCE (BV-RMCE) to replace the *eGFP* gene with the *mCherry* gene in transgenic U87 cells. (**A**) Fluorescence images to demonstrate the transition of EGFP expression to mCherry expression by Cre-RMCE at different time points. (**B**) Flow cytometry analysis to show the change from EGFP-positive U87 cells at day 0 to mCherry-positive cells at day 30. (**C**) PCR genotyping to confirm *AAVS1* integration of the *mCherry* cassette. A primer specific for the *mCherry* gene and a primer specific for chromosome 19 downstream of the 3′-end of the right *AAVS1* homologous arm were used. The amplification of a 3-kb fragment demonstrates the successful cassette exchange at *AAVS1* through BV-RMCE.

After the transgenic iPSC colonies selected from TALEN-mediated transgene insertion were co-transduced with BV-Cre and BV-mCherry at an MOI of 100 pfu per cell each, we observed that the original *floxed eGFP* donor cassette at the *AAVS1* locus could be exchanged with the *mCherry* donor cassette flanked by the same heterospecific *loxP* sequences (Figure 4B). Hygromycin B-selection was used to enrich mCherry-positive iPSCs. Most of the cells died off in the first week of the selection. Small colonies remained in the second week, which expanded in the third and forth weeks to form pure mCherry-positive iPSC colonies. When genomic DNA of the hygromycin B-resistant transgenic iPSCs was subjected to PCR genotyping using a primer specific for the *mCherry* gene present in the donor cassette and a primer specific for chromosome 19 downstream of the 3′-end of the right homologous arm, the amplification of a 3-kb fragment confirmed the successful site-specific integration of the *mCherry* transgene within the *AAVS1* locus by Cre-RMCE (Figure 4C). Immunostaining of the mCherry-positive iPSC colonies confirmed the expression of the pluripotent markers, SSEA4, SOX2, NANOG, Tra-1-60 and OCT3/4 (Figure 4D), indicating that the cells retained their pluripotency property after baculoviral vector-mediated Cre-RMCE. We also tested TALEN-modified EGFP-positive U87 cells and observed successful Cre-RMCE by baculoviral transduction, as shown by the stable expression of mCherry in the U87 cells after hygromycin B-selection (Figure 5).

## DISCUSSION

Human iPSCs, like ESCs, are able to develop into many different cell types. Progenies derived from iPSCs can be used as transplantable cells for tissue repairing and regeneration in regenerative medicine. Such medical applications would benefit greatly from constant and steady expression of functional transgenes after the genes are integrated into a selected genome locus in iPSCs. Progenies derived from genetically modified iPSCs can also be used for gene therapy purpose to deliver therapeutic gene products. For example, tumor-tropic neural stem cells or mesenchymal stem cells can be generated by differentiation of genetically engineered human pluripotent stem cells and used for tumor-targeted gene therapy.

Retrovirus-mediated random integration is currently commonly used for gene addition in hESCs, iPSCs and their progenies for stable expression of a transgene. However, random integration can result in insertional mutagenesis, oncogenic activation and subsequently tumorigenesis of transplanted cells (32,33). Furthermore, expression of a randomly integrated transgene is unpredictable. Integration within certain DNA sequence contexts can trigger epigenetic silencing causing variegated expression in clonally isolated cells or occurrence of transgene expression cessation either over time or on differentiation (26,34–36). Hence, there is a compelling need to develop techniques to integrate transgene into a defined genomic locus that allows stable expression of the gene during expansion and differentiation of ESCs and iPSCs in the absence of genome mutagenesis. TALEN-mediated gene targeting is one of the most promising candidate technologies that meet this requirement.

TALEN-targeting systems have recently generated much excitement and been widely adopted for HR-mediated genetic modificaiton, with significantly increased targeting efficiency as compared with conventional HR (4,11–13,16). A previous study reported by Hockemeyer *et al.* (7) used electroporation to deliver customized designed TALENs and a donor plasmid and has shown that ∼50% human ESC colonies and iPSC colonies were *AAVS1*-targeted, and ∼20 site-specifically modified colonies could be picked from 10^7^ cells. In the present study, we have used TALENs with a new specificity to target a novel site in *AAVS1*. The single-strand annealing assay in yeast and T7EI endonuclease assay in human cells demonstrated that the cleavage rate of the TALENs using in current study was excellent (Supplementary Figure S1, Figure 1B). Using baculoviral transduction in human cells, we observed that the targeted integration efficiency mediated by BV-TALENs was close to 100%, and at least 20 colonies could be picked up from 2 × 10^4^ U87 cells (Table 1). Thus, compared with other transgene delivery systems, BV-TALEN appears more efficient for targeted integration, which possibly benefits from high transduction efficiency of baculoviruses. When genome toxicity associated with different dosages of BV-TALEN was evaluated, we observed pronouncedly increased number of DNA DSB only when an MOI above 200 pfu per cell was used, indicating that the dose of BV-TALEN used in the current study (MOI = 100) was not only efficient in inducing TALEN-mediated HR in human iPSCs but also safe.

Targeted integration efficiency is also related to the size of DNA insert (37). Inserting multiple transgenes or a large expression cassette into a targeted genome site with high targeting efficiency remains a hurdle for genetic engineering, but it is practically important and useful. For example, insertion of multiple reprograming transcript factors into a defined human genome site could be used for somatic cell reprograming to generate iPSCs. A previous study has successfully inserted a 7.7 kb cassette into mammalian genome with HR integration by ZFNs (37). It was unclear whether a DNA insert larger than this could be integrated into human genome with ZFN or TALEN technology. Using baculoviral vectors as a gene delivery system in the present study, we demonstrated for the first time that TALEN-mediated HR could integrate DNA insert up to 13.5 kb (Figure 2E). Hence, owing to the large cloning capacity of baculoviral vectors, TALEN-mediated HR by baculoviral transduction could be an attractive option for site-specific genome editing with a large donor cassette.

Baculoviral transduction has been used to express ZFNs and deliver a DNA donor template for site-specific editing of the *CCR5* locus in human ESCs (23). Adeno-associated virus-based vector platforms have also been successfully used for ZFN gene delivery into human cells (38,39). Unlike ZFN technology, TALEN delivery systems tested so far are basically are non-viral in nature. The design architecture of TALENs is different from the one for ZFNs and contains repeated sequences in RVDs. Viruses are often prone to genetic recombination when repeated sequences are present. This may make it difficult to generate effective TALEN-expressing viral vectors. Baculoviruses have double-stranded DNA genomes that are covalently closed circles. Baculovirus genomes vary in size between 80 and 180 kb, in which only a set of 29 core genes are conserved, and most are multiple homologous regions with repeated sequences (40). As evidenced from the genomic variations within baculoviruses isolates from the field, baculovirus genomes hold a certain degree of plasticity. These features of baculovirus may explain our observation in the current study that TALEN-repeated sequences in a baculoviral vector do not obviously affect overall TALEN activity, and BV-TALEN is still effective in mediating genetic modification.

Although highly efficient in mediating site-specific integration, TALEN technology introduces chromosomal DSB at a target site, possibly also off-target DNA breaks at related sequences throughout the genome. Both could lead to potentially hazardous effects. At high ZFN concentrations, activation of the nuclease that cleaves at non-intentional sites may occur due to ZFN dimerization in the absence of DNA binding at related sequences (41). It remains unclear whether similar activation may occur at high TALEN concentrations. Therefore, the ZFN and TALEN procedure used for genetic modification of iPSCs would be complicated in clinical applications, as extensive screening is required to select an iPSC clone without obvious genomic damage. We envision that an iPSC clone with *loxP* sites that has passed genome toxicity screening after ZFN or TALEN treatment could be used as a genetically amenable master cell line for readily and safely introducing different *floxed* transgenes into a predefined site, e.g. *AAVS1*, through Cre-RMCE. In the present study, we first used TALENs to introduce mutually incompatible heterospecific *loxP* sites into the *AAVS1* locus. The use of the heterospecific *loxP* sequences, as opposed to wild-type *loxP* sequences, was to avoid intra-molecular recombination and to ensure that only unidirectional transgene integration is permitted within the *AAVS1* locus. We demonstrated that by using two baculoviral vectors, one acting as a transgene donor and another as source of Cre expression, an original transgene cassette with the heterospecific *loxP* sites was efficiently replaced by another transgene cassette with the same *loxP* sites in iPSCs, without affecting pluripotency. This RMCE strategy can be adopted to build versatile transgenic iPSCs with inducible transgene expression, lineage-specific reporter and constitutive functional gene expression, as already tried in human ESCs (42).

In conclusion, we have developed a novel and robust gene editing tool using BV-TALEN coupled with BV-Cre/loxP. This baculovirus-based combined approach offers the advantage of high genome-targeted editing efficiency with low genome toxicity and allows repeatable yet accurate insertion of different transgene cassettes into a predefined genomic site in a master iPSC line with a pair of heterospecific *loxP* sequences. Its potential is well-worth exploring further, especially in engineering human pluripotent stem cells for biological and biomedical applications.

## SUPPLEMENTARY DATA

Supplementary Data are available at NAR Online.

## FUNDING

The Singapore Ministry of Health’s National Medical Research Council [NMRC/1284/2011]; the Singapore Ministry of Education [MOE2011-T2-1-056]; Singapore Agency for Science, Technology and Research Joint Council [11/03/FG/07/02] and Institute of Bioengineering and Nanotechnology (Biomedical Research Council, Agency for Science, Technology and Research, Singapore). Funding for open access charge: [NMRC/1284/2011].

*Conflict of interest statement*. None declared.

## Supplementary Material

Supplementary Data
