# Supplementary material for: Baculoviral transduction facilitates TALEN-mediated targeted transgene integration and Cre/LoxP cassette exchange in human-induced pluripotent stem cells
Source: Nucleic Acids Res. 2013 Aug 13;41(19):e180. doi: 10.1093/nar/gkt721 (PMC3799456; doi:10.1093/nar/gkt721)
Supplement: Supplementary Data [file supp_41_19_e180__index.html]

Baculoviral transduction facilitates TALEN-mediated targeted transgene integration and Cre/LoxP cassette exchange in human-induced pluripotent stem cells — Baculoviral transduction facilitates TALEN-mediated targeted transgene integration and Cre/LoxP cassette exchange in human-induced pluripotent stem cells — Supplementary Data 

# Baculoviral transduction facilitates TALEN-mediated targeted transgene integration and Cre/LoxP cassette exchange in human-induced pluripotent stem cells

## Supplementary Data

files

**Files in this Data Supplement:**

- Supplementary Data - pdf file
