# Supplementary material for: Baculoviral transduction facilitates TALEN-mediated targeted transgene integration and Cre/LoxP cassette exchange in human-induced pluripotent stem cells
Source: Nucleic Acids Res. 2013 Aug 13;41(19):e180. doi: 10.1093/nar/gkt721 (PMC3799456; doi:10.1093/nar/gkt721)
Supplement: Supplementary Data [file supp_gkt721_nar-00740-met-g-2013-File007.pdf]

Baculoviral Transduction Faciliates TALEN-Mediated Targeted Transgene Integration and Cre/LoxP Cassette Exchange in Human Induced Pluripotent Stem Cells

Haibao Zhu,[1#] Cia-Hin Lau,[1#] Sal-Lee Goh,[1] Qingle Liang,[1] Can Chen,[1] Shouhui Du,[1] Rui-Zhe Phang,[1] Felix Chang Tay,[1] Wee-Kiat Tan,[1] Zhendong Li,[1] Johan Chin-Kang Tay,[1] and Shu Wang[1,2]*

[1]Department of Biological Sciences, National University of Singapore, Singapore
[2]Institute of Bioengineering and Nanotechnology, Singapore

SUPPLEMENTARY INFORMATION

[#] The two authors contributed equally to the work.

*Corresponding author:
Shu Wang, Ph.D.
Department of Biological Sciences, National University of Singapore, Singapore 117543
Telephone: 65-6874-7712; Fax: 65-6779-2486
e-mail: dbsws@nus.edu.sg

Supplementary Table

Table S1: Primers used in the current study

Primer Sequence<br>(Forward, FP; Reverse, RP)	Amplicon Size<br>(bp)	Description
FP (AAVS1_SnaBI):<br>5'-GCGC TACGTA CTGAACCTGAGCCAGCTCCCATA-3'<br>RP (AAVS1_SalI):<br>5'-ATATGTCGAC GAAGACTAGCTGAGCTCTCGGACC-3'	864	Construct left homologous arm
FP (AAVS1_NotI):<br>5'-ATATGCGGCCGC CCCTATGTCCACTTCAGGACAGCA-3'<br>RP (AAVS1_BstBI):<br>5'-GCGC TTCGAATGCCAAGGACTCAAACCCAGAAG-3'	879	Construct right homologous arm
FP (EGFP):<br>5'-GCGACGTAAACGGCCACAAGTT-3'<br>RP (AAVS1):<br>5'-GCCTCCCTAAGACCCAGAAGTCCAG-3'	2886	Check for site-specific integration of EGFP donor
FP (WPRE):<br>5'-GCTACGTCCCTTCGGCCCTCAATC-3'<br>RP (AAVS1):<br>5'-GCCTCCCTAAGACCCAGAAGTCCAG-3'	3038	Check for site-specific integration of 4F-EGFP donor
FP (EGFP):<br>5'-GCGACGTAAACGGCCACAAGTT-3'<br>RP (EGFP):<br>5'-CTGGGTGCTCAGGTAGTGGTTG-3'	554	Design probe for Southern blot
FP (ACTB):<br>5'-TGGCACCCAGCACAATGAAG-3'<br>RP (ACTB):<br>5'-GATGGAGGGGCCGGACTC-3'	146	Actin
FP (Oct4):<br>5'-GAGCAAAACCCGGAGGAGT-3'<br>RP (Oct4):<br>5' -TTCTCTTTCGGGCCTGCAC-3'	310	Oct4 pluripotency marker
FP (Sox2):<br>5'- GGGAAATGGGAGGGGTGCAAAA-3'<br>RP (Sox2):<br>5'- TTGCGTGAGTGTGGATGGGATT-3'	151	Sox2 pluripotency marker
FP (Nanog):<br>5' -GCTTGCCTTGCTTTGAAGCA-3'<br>RP (Nanog):<br>5' -TTCTTGACCGGGACCTTGTC-3'	256	Nanog pluripotency marker
FP (NELF):<br>5'-AAGCGTAATTCTGTTGCCTCAAG-3'	196	NELF ectoderm marker

RP (NELF):<br>5’-GTCCAACCAGTCAAGCTATCATTG-3’		
FP (Pax6):<br>5’-CAATAATGTTGACGGTGACTATC-3’<br>RP (Pax6):<br>5’-AGAAGGAAGCGACACTCTG-3’	179	Pax6 ectoderm marker
FP (α-MHC):<br>5'-GTCATTGCTGAAACCGAGAATG-3'<br>RP (α-MHC):<br>5'-GCAAAGTACTGGATGACACGCT-3'	413	α-MHC mesoderm marker
FP (PPAR-γ):<br>5’-ATTGACCCAGAAAGCGATTC-3’<br>RP (PPAR-γ):<br>5’-CAAAGGAGTGGGAGTGGTCT-3’	154	PPAR-γ mesoderm marker
FP (AL133):<br>5’-ACTGCTGTTGATGTCTTTCTGTG-3’<br>RP (AL133):<br>5’-CGTACACGTCCTCCGAATCC-3’	157	AL133 endoderm marker
FP (AFP):<br>5’-CCCGAACTTTCCAAGCCATA-3’<br>RP (AFP):<br>5’-TACATGGGCCACATCCAGG-3’	101	AFP endoderm marker
FP (mCherry):<br>5’-CAACACGCGTCTATGGTGAGCAAGGGCGA-3’<br>RP (AAVS1):<br>5’-GCCTCCCTAAGACCCAGAAGTCCAG-3’	3003	Check for site-specific Cre-LoxP cassette exchange of mCherry donor
FP(off-target 1)<br>5’-TTCTTCGTGGTTCCCACCAG-3’<br>RP(off-target 1)<br>5’-TGATCTACCTGGGGCTTTGC-3’	337	Off-target site 1
FP(off-target 2)<br>5’-AAGAAGACGGTTGTTGGAAAC-3’<br>RP(off-target 2)<br>5’-GTTTATCCTAGGAAGGCAATGTTAT-3’	440	Off-target site 2
FP(off-target 3)<br>5’- CTTTGAGGCTAAGGGGTCTG-3’<br>RP(off-target 3)<br>5’- AGAAGGGCTGAGCACTGGTG -3’	353	Off-target site 3
FP(off-target 4)<br>5’- TGACATCCAATGGCACCCAA -3’<br>RP(off-target 4)<br>5’- CAGAGCACACATTCAGGGGT -3’	404	Off-target site 4
FP(off-target 5)<br>5’- AATGAGCTCGTAGACCTGCC -3’<br>RP(off-target 5)	218	Off-target site 5

5’- AGTGGCTTCCCAAAGATGGAA -3’		
FP(off-target 6)<br>5’- TGGGAGTAGCATTTTCGGCA -3’<br>RP(off-target 6)<br>5’- AGAACGTCCCAGCAAGACAG-3’	335	Off-target site 6
FP(off-target 7)<br>5’- GCAAGAGCACACTTTCCAGC -3’<br>RP(off-target 7)<br>5’- TCTGATGACCCCTCGGTGTA -3’	337	Off-target site 7
FP(off-target 8)<br>5’- TGAAAGTTGGCCCCAGAGTC-3’<br>RP(off-target 8)<br>5’- ACTAATGGAGCACATGGCCG -3’	294	Off-target site 8
FP(AAVS1)<br>5’- TCTAACGCTGCCGTGCCGTCTCTCTCCTGA -3’<br>RP(AAVS1)<br>5’- CGGGGATGCAGGGGAACGGGGCTCAGTCTG -3’	1600	Remaining allele deletion

Table S2: Eight BLAST-predicted genomic off-target (OT) sites

Rank	Chr	Location	Sequence	Gap(bp)	Matched bp
AAVS1	19	28116242	CCGAGAGCTCAGCTAGTCTTCTTCC TCCAACCCGGGCCCCTATGTCC	17	L 15/15; R 15/15
OT1	11	7679322	GCCCTCACCTCAGCCTCCTGGAGGGCCA CCAACCCGGGCCCCCACTGTACCA	16	L 10/15; R 13/15
OT2	8	11331521	TCCTCCTTTGCAGCCAGTCTTCTTCCTCC ATCCCAGGCCCAGACAGTCA	14	L 7/15; R 10/15
OT3	11	11484751	AGACTGAGCTCAGCTCGTCTTCTTCCTG GACCTTGGACTCCTTGGCAGA	19	L 10/15; R 9/15
OT4	1	4408187	TCCAGGTGCGTGCAGGGACAGGGCTG GGCCCCTGGGGAGGGGCCCCTATGTCCA	21	L 7/15; R14/15
OT5	17	26934696	GTGGCAGAAGACAGCCACTGTTCTGTTGG TCAGCAGGGCATGTGCCTTTGTTTT	18	L 8/15; R10/15
OT6	9	37382578	TAAGAATTCTTTTCTGGGATTGTTACTTT AGTCCCTGGGTGAAAGGAGGA	23	L 6/15; R 6/15
OT7	17	44866370	GCTGAGAATTCTAGATTGAGAAACTAT GTGGAGGTAACACCTGTGCA	26	L 7/15; R 6/15
OT8	1	19097242	TGAGCAGAGCTCAGCTAGTGACCCTCT CAGGAAGGCGACGAGGAAGA	19	L 12/15; R 4/15

L: The sequence for left binding domain; R: The sequence for right binding domain.

Supplementary Figures

Figure S1. Single strand annealing (SSA) assay for determination of TALEN cleavage rate in yeast. The assay was performed using custom TALEN service provided by Cellectis Bioresearch (Paris, France). (**A**) An overview of the SSA assay process. A first haploid strain of yeast expresses a plasmid encoding TALEN sequences while a second haploid strain of yeast expresses a plasmid carrying a non-functional LacZ gene. The LacZ gene is interrupted by an internal duplication of 700 bp to 220 bp while TALEN recognition site is integrated between the two repeats. Upon cleavage with TALEN, the double-strand break is repaired by single-strand annealing. The restoration of a functional LacZ gene allows expression of the β-galactosidase enzyme that can be detected by a blue-white assay. (**B**) A blue/white colorimetric assay to determine the TALEN cleavage rate in yeast. The β-galactosidase activity is directly associated with the homologous recombination efficiency. The cleavage rate of the TALENs detected in the assay was 87%.

Figure S2. PCR genotyping to confirm TALEN-mediated, *AAVS1*-specific transgene integration in human U87 cells. A primer specific for the *eGFP* gene present in the DNA donor BV-eGFP and a primer specific for chromosome 19 downstream of the 3' end of the right homologous arm (see Fig. 1) were used. The amplification of a 2.9 kb fragment demonstrates the successful *AAVS1* modification through BV-TALEN mediated HR. Including results in Fig. 2C, totally 41 clones were examined.

Figure S3. Southern blot analysis to detect the modified *AAVS1*. Five transgenic clones were examined after digestion with ApaLI and hybridization. Single 12 kb fragment (arrow) was detected in these clones. Including results in Fig. 2D, totally 8 clones were examined.

Figure S4. Analysis of NHEJ mutations in U87 cells. The genomic off-target sites were amplified from genomic DNA isolated from wild-type (WT) U87 and three AAVS1-targeted clones generated in this study. DNA sequencing was performed to examine NHEJ mutations at eight BLAST-predicted genomic off-target (OT) sites (Table S2). Chromatograph results are presented. The reference sequences for each OT site are listed on the top.

Figure S5. Karyotype diagram of transgenic EGFP-positive human iPSCs. Homologous pairs of chromosomes are identified by their general shape, length, and the pattern of banding produced by a Giemsa staining. Results from 6 iPSCs are shown.

Figure S1

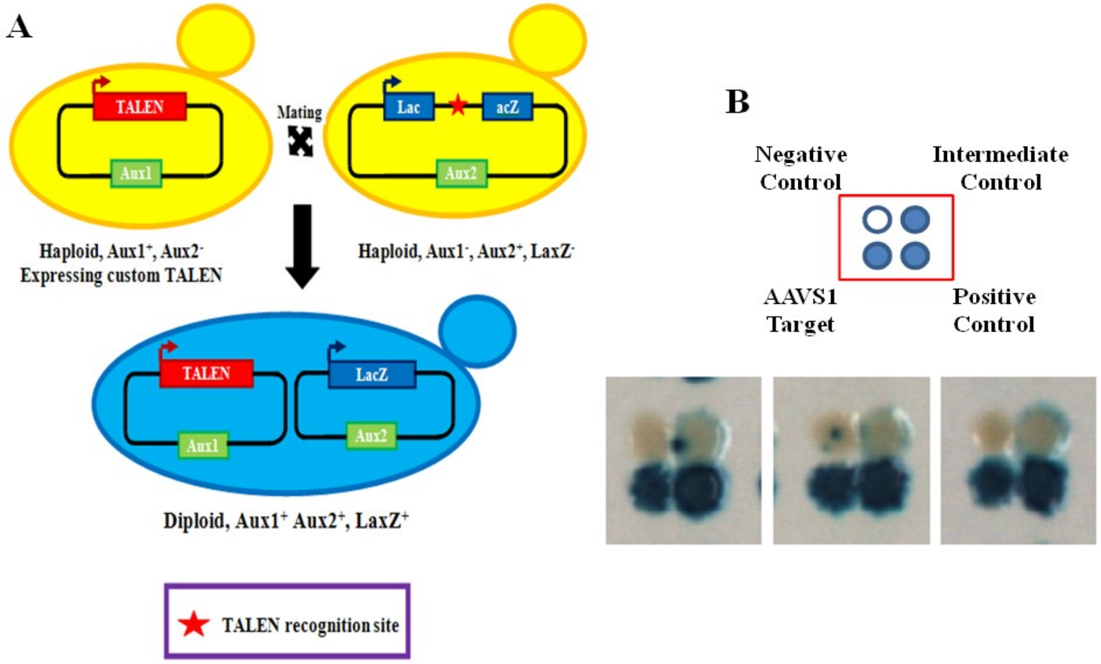
A
TALEN
Aux1
Mating
Lac
acZ
Aux2
Haploid, Aux1+, Aux2-
Expressing custom TALEN
Haploid, Aux1-, Aux2+, LaxZ-
TALEN
LacZ
Aux1
Aux2
Diploid, Aux1+ Aux2+, LaxZ+
TALEN recognition site
B
Negative
Control
Intermediate
Control
AAVS1
Target
Positive
Control

Figure S2

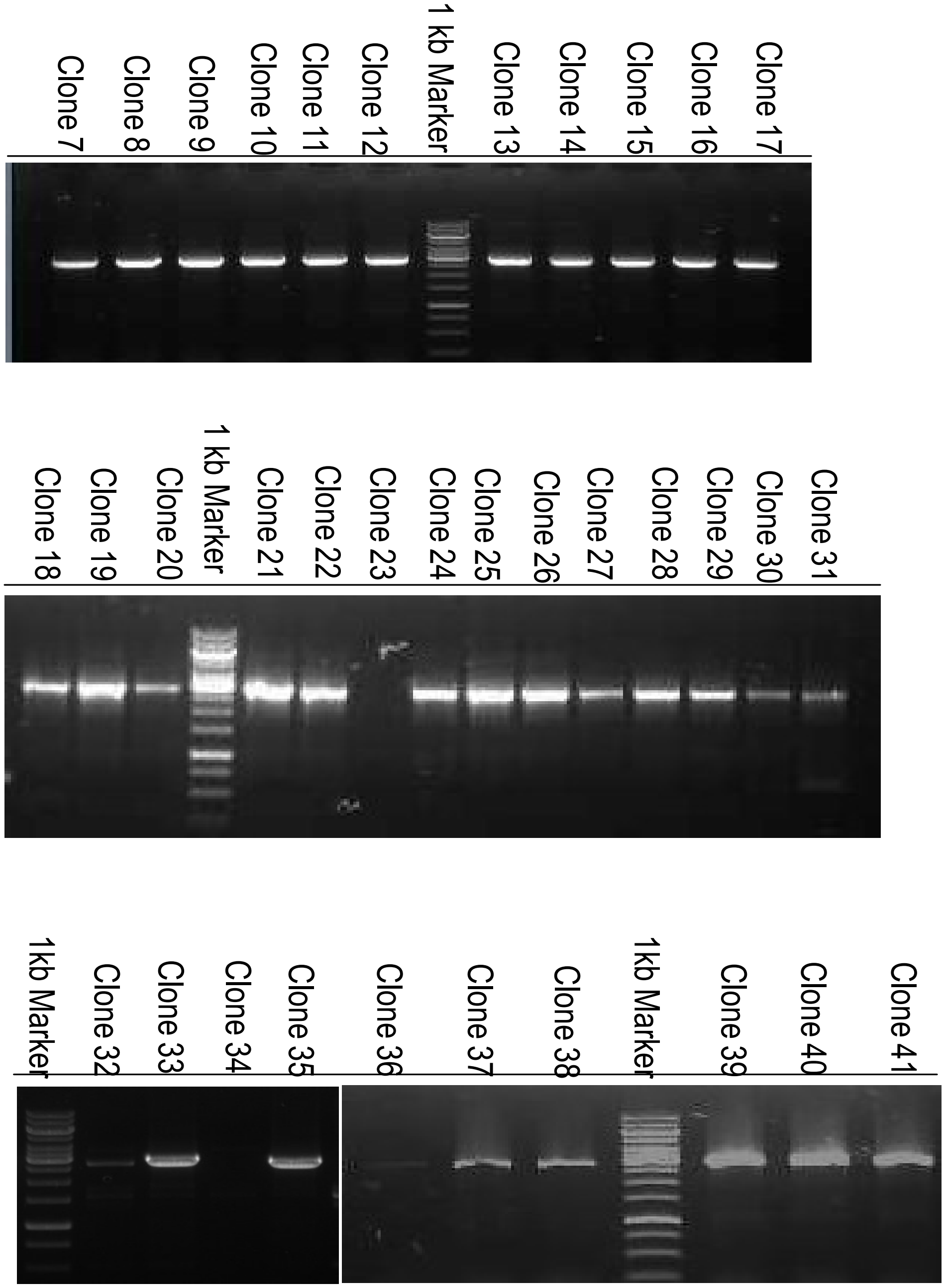
Clone 7
Clone 8
Clone 9
Clone 10
Clone 11
Clone 12
1 kb Marker
Clone 13
Clone 14
Clone 15
Clone 16
Clone 17
Clone 18
Clone 19
Clone 20
1 kb Marker
Clone 21
Clone 22
Clone 23
Clone 24
Clone 25
Clone 26
Clone 27
Clone 28
Clone 29
Clone 30
Clone 31
1kb Marker
Clone 32
Clone 33
Clone 34
Clone 35
Clone 36
Clone 37
Clone 38
1kb Marker
Clone 39
Clone 40
Clone 41

Figure S3

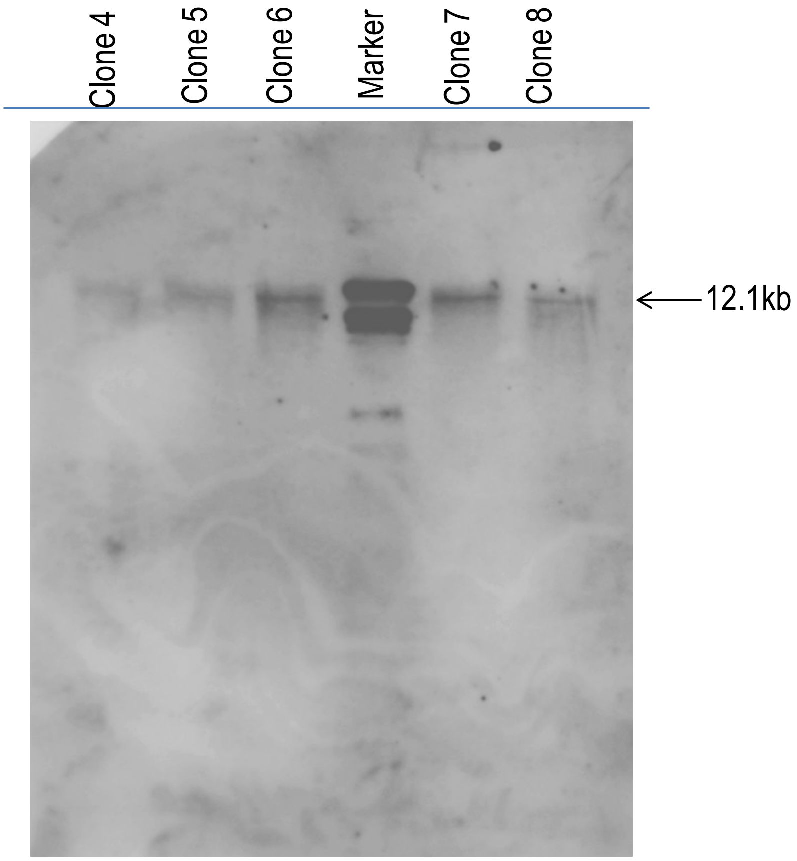

Figure S4

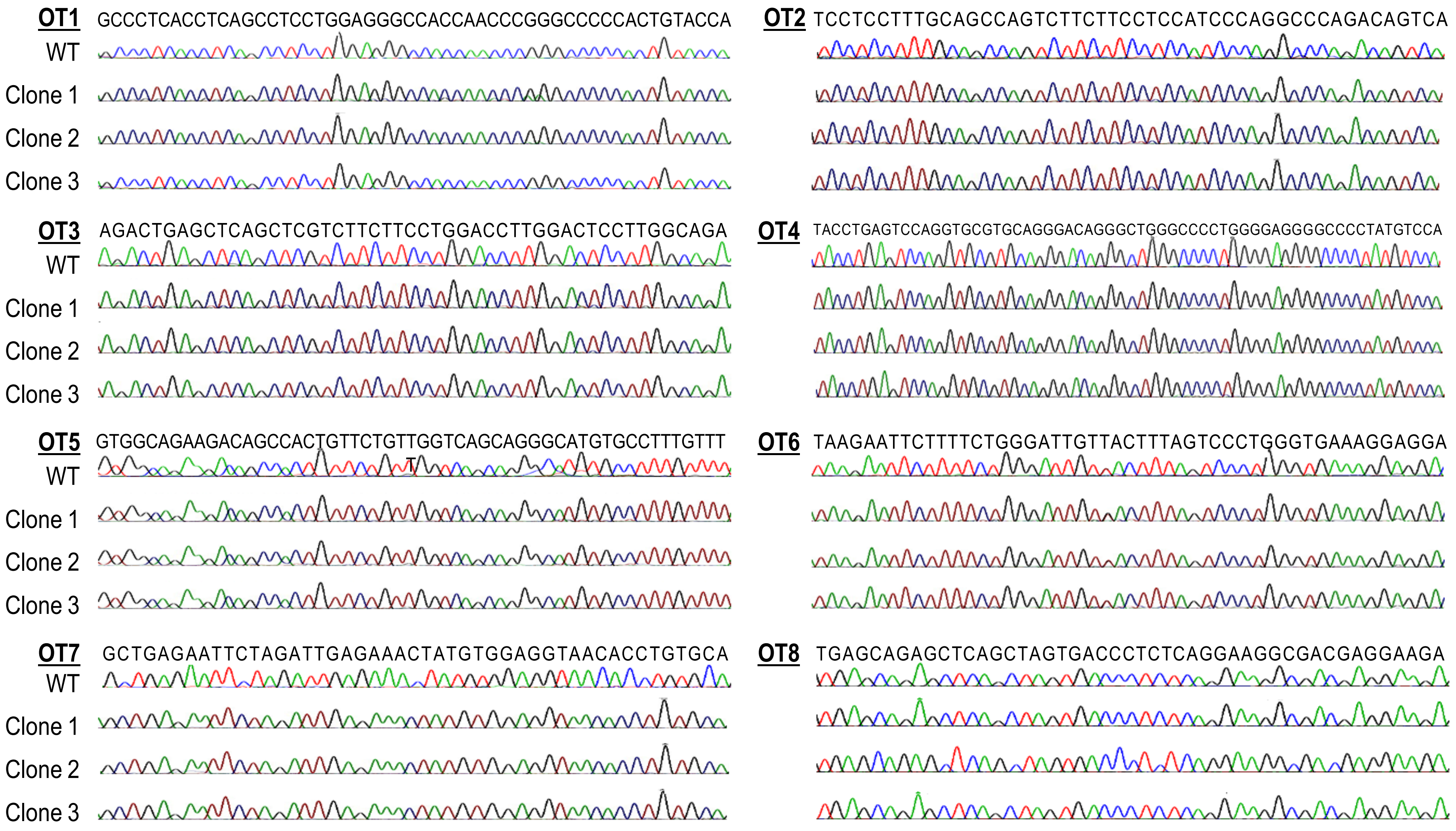
OT1 GCCCTCACCTCAGCCTCCTGGAGGGCCACCAACCCGGGCCCCCACTGTACCA
WT
Clone 1
Clone 2
Clone 3
OT2 TCCTCCTTTGCAGCCAGTCTTCTTCCTCCATCCCAGGCCCAGACAGTCA
OT3 AGACTGAGCTCAGCTCGTCTTCTTCCTGGACCTTGGACTCCTTGGCAGA
WT
Clone 1
Clone 2
Clone 3
OT4 TACCTGAGTCCAGGTGCGTGCAGGGACAGGGCTGGGCCCCTGGGGAGGGGCCCCTATGTCCA
OT5 GTGGCAGAAGACAGCCACTGTTCTGTTGGTCAGCAGGGCATGTGCCTTTGTTT
WT
Clone 1
Clone 2
Clone 3
OT6 TAAGAATTCTTTTCTGGGATTGTTACTTTAGTCCCTGGGTGAAAGGAGGA
OT7 GCTGAGAATTCTAGATTGAGAAACTATGTGGAGGTAACACCTGTGCA
WT
Clone 1
Clone 2
Clone 3
OT8 TGAGCAGAGCTCAGCTAGTGACCCTCTCAGGAAGGCGACGAGGAAGA

Figure S5

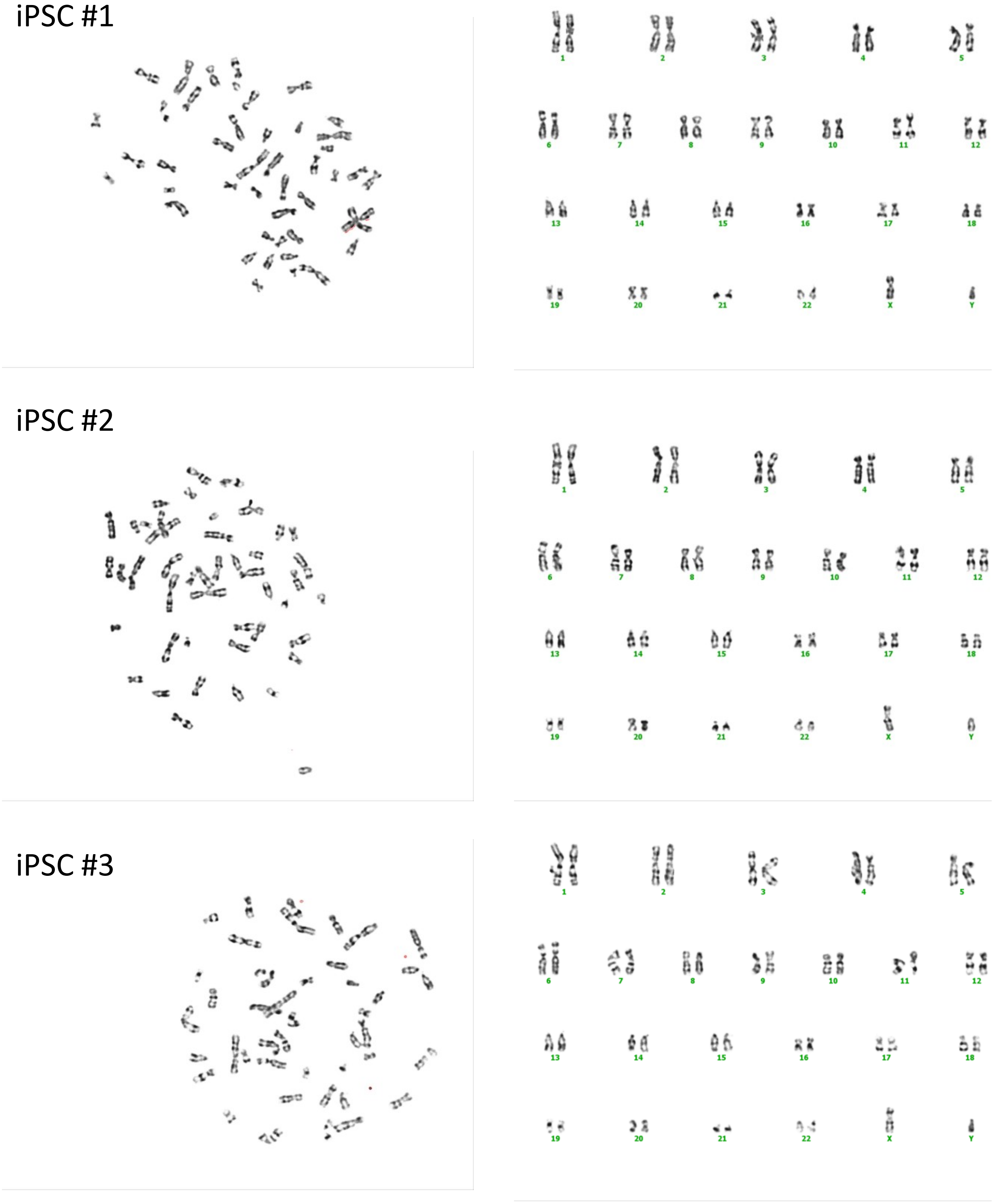

Figure S5

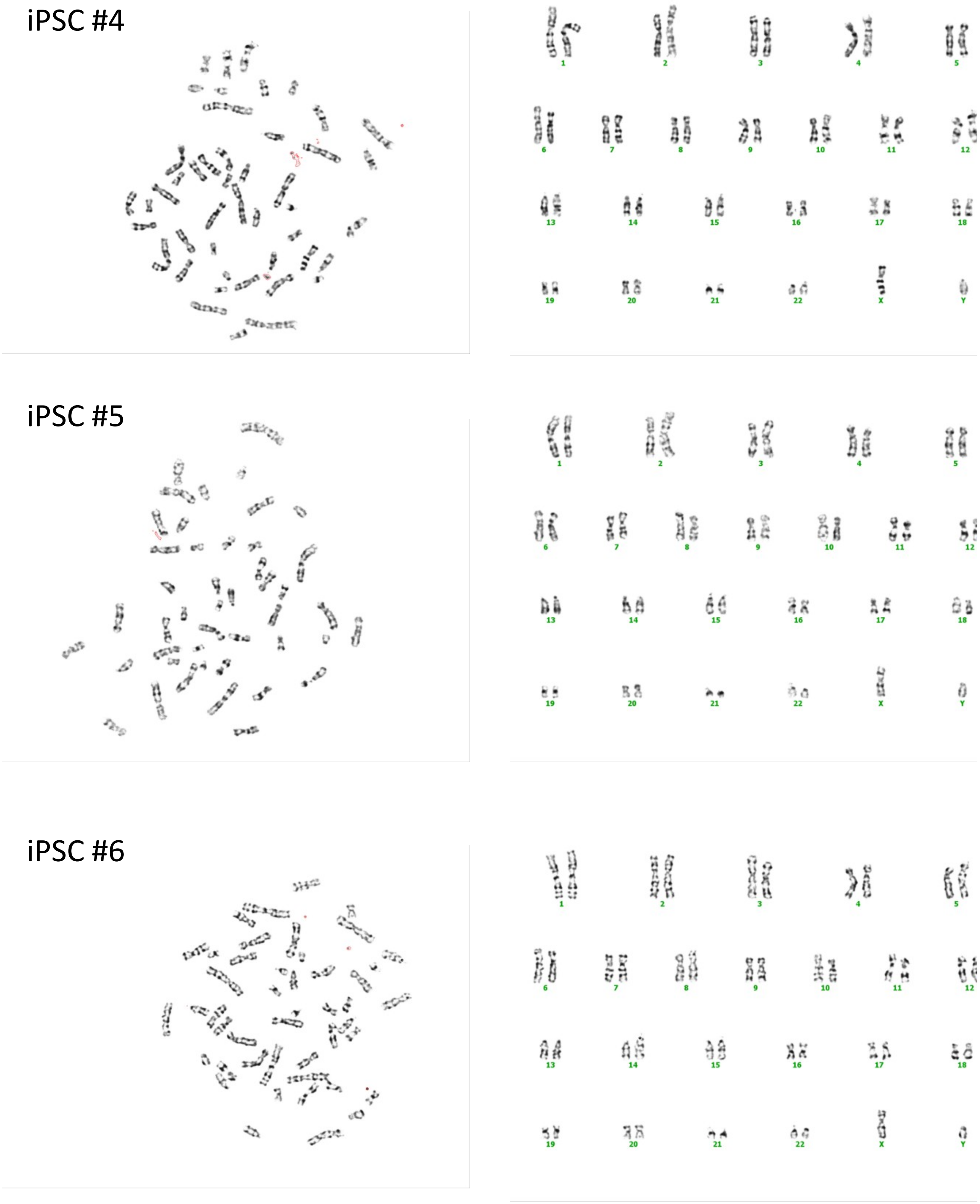

iPSC #4
iPSC #5
iPSC #6
1 2 3 4 5
6 7 8 9 10 11 12
13 14 15 16 17 18
19 20 21 22 X Y
